# Investigación en cáncer en el contexto de la pandemia de la enfermedad por SARS-CoV-2

**Published:** 2020-06-30

**Authors:** Carolina Wiesner

**Affiliations:** 1 Directora, Instituto Nacional de Cancerología, Bogotá, D.C., Colombia Instituto Nacional de Cancerología BogotáD.C. Colombia

En diciembre de 2019, en Wuhan (China), se reportaron los primeros casos del síndrome respiratorio agudo grave *(Severe Acute Respiratory Syndrome,* SARS) causado por un coronavirus (CoV), síndrome que se denominó SARS COVID-19 (COV: coronavirus; ID: *infectiuous disease).* La infección por este virus que se transmite de persona a persona, se propagó rápidamente desde China a otros países y otros continentes, de manera que el 11 de marzo de 2020 la OMS declaró el estado de pandemia en la cual nos encontramos actualmente ^1^. En la Región de las Américas, las cifras de casos infectados hasta el 23 de mayo eran de 3.204 casos en Honduras y de 1'547.973 en los Estados Unidos, en tanto que en Brasil se registraban 310.000 casos y, en Colombia, 18.330 ^2^.

Al observar la variabilidad registrada en las tasas de infección y de letalidad han aparecido muchas teorías basadas en diversos aspectos, como las diferencias en la estructura poblacional de los países, las medidas nacionales y regionales tomadas por los gobiernos en los diferentes momentos de la pandemia, la capacidad y la oportunidad del reporte de los resultados de las pruebas moleculares de diagnóstico de COVID-19, las características y la organización de los sistemas de salud, el acceso a los servicios, y la prevalencia de los factores de riesgo *y* de enfermedades crónicas, entre otros ^3^^,^^4^. Ante este panorama, han surgido múltiples preguntas de investigación, clasificadas por la OMS en cerca de 90 temáticas ^5^ y_,_ aunque los estudios sobre cáncer y COVID-19 no son los más numerosos (0,23 %) ^5^, sin lugar a dudas han ido en aumento en la medida en que los centros hospitalarios avanzan en el trabajo articulado.

En el marco de la pandemia, los pacientes con cáncer enfrentan un reto importante para acceder al diagnóstico oportuno y el tratamiento multidisciplinario y continuo en busca de su supervivencia ^6^. Esta situación se ha visto afectada negativamente por las medidas de distanciamiento social, la cuarentena poblacional, y la preparación de los centros hospitalarios públicos y privados para la atención de los pacientes con COVID-19 ^6^^,^^7^. Asimismo, ha quedado claro que un paciente con cáncer y positivo para el SARS-CoV-2 debe modificar o diferir su tratamiento oncológico hasta que la infección ceda, por lo que en algunos países se ha registrado o estimado un aumento excesivo de la mortalidad por cáncer ^8^^-^^10^.

En esta situación de pandemia de COVlD-19, también se plantean muchas preguntas de investigación en torno al cáncer ¿qué impacto tienen las medidas poblacionales de control de la transmisión de COVID-19 en la incidencia del cáncer, el estadio de la enfermedad en el momento del diagnóstico, la supervivencia global y el exceso de mortalidad?, ¿cuál es la prevalencia de la infección asintomática y de la sintomática en los pacientes con cáncer según los grupos de edad?, ¿cuál es el diagnóstico diferencial entre esta y otras infecciones concomitantes en los pacientes con cáncer sospechosos de tener COVID-19?, ¿cuáles son las particularidades de la dinámica de la infección según el tipo de cáncer y la edad?, ¿cuáles son las diferencias y los factores asociados con la propensión a la infección y su gravedad en los pacientes con cáncer?, ¿cómo se afectan los resultados clínicos al modificar los esquemas terapéuticos?

La prevalencia de la infección hospitalaria en los pacientes con neoplasia maligna ha oscilado entre 0,7 % en China y 6 a 8 % en Nueva York e Italia, en tanto que, en los niños menores de 10 niños, ha sido del 1 % en Nueva York ^11^^-^^14^. Preocupa, además, la prevalencia de la infección asintomática en los pacientes con cáncer, reportada en 4 % ^15^, especialmente antes de los procedimientos quirúrgicos o inmunosupresores, dado su mayor riesgo de desarrollar comorbilidades graves, con el consecuente aumento de la mortalidad en el contexto de la COVID-19 ^15^^,^^16^.

En Colombia, no se ha recomendado practicar la prueba molecular de diagnóstico antes de los procedimientos quirúrgicos en los pacientes asintomáticos ^17^. Sin embargo, es importante evitar el daño a los pacientes con cáncer e infección por SARS-CoV-2 al iniciar un tratamiento oncológico, por el riesgo de aumentar la morbilidad o la mortalidad. La tasa de letalidad en pacientes con cáncer y COVID-19 fue de 28,6 % en China según un estudio ^18^ y, en otro más amplio de cohorte, fue de 13 % ^16^ comparado con la tasa en pacientes sin cáncer, que fue de 2,3 % ^19^. Es por ello que, recientemente, se han emitido recomendaciones que proponen la tamización ^6^^,^^19^^,^^20^. En este sentido, el Instituto Nacional de Cancerología ha iniciado la elaboración de un protocolo de estudio para resolver este aspecto.

La pandemia de COVID-19 ha planteado múltiples retos para la atención en salud, así como preguntas de investigación sobre el cáncer en el marco de una profunda transformación de la sociedad en su conjunto. Se ha generado, además, un espacio de reflexión sobre los efectos de la globalización, así como de las capacidades de los sistemas de ciencia, tecnología e innovación y de salud en Colombia. Sin duda, en este contexto se hace más explícita la necesidad de articular los dos sistemas, con el fin de fortalecer las capacidades de generar conocimientos y garantizar la disponibilidad de tecnologías acordes con las necesidades y características locales. La pandemia nos enfrenta, asimismo, al desafío del control diferencial de las enfermedades agudas en su relación con las crónicas.

Sea esta la oportunidad para hacer un reconocimiento al Instituto Nacional de Salud por el papel protagónico que ha tenido en el marco de esta pandemia. Esperamos que el Estado comience a dar respuesta a las necesidades transversales y estructurales de las instituciones de ciencia y tecnología y de prestación de servicios de salud, para garantizar el cabal cumplimiento de sus funciones y potencialidades técnicas, humanas y científicas. Este momento de crisis pone de manifiesto la necesidad imperativa de fortalecerlas con pleno reconocimiento de su valor público.
